# Association of Serum Vitamin C With NAFLD and MAFLD Among Adults in the United States

**DOI:** 10.3389/fnut.2021.795391

**Published:** 2022-02-04

**Authors:** Zhi-Qin Xie, Hong-Xia Li, Wen-Liang Tan, Lei Yang, Xiao-Wu Ma, Wen-Xin Li, Qing-Bin Wang, Chang-Zhen Shang, Ya-Jin Chen

**Affiliations:** ^1^Department of Hepatobiliary Surgery, Sun Yat-sen Memorial Hospital, Sun Yat-sen University, Guangzhou, China; ^2^Department of Pathology, The First Affiliated Hospital, Sun Yat-sen University, Guangzhou, China; ^3^Department of Cardiology, The Eighth Affiliated Hospital, Sun Yat-sen University, Shenzhen, China

**Keywords:** non-alcoholic fatty liver disease, liver fibrosis, liver cirrhosis, vitamin C, metabolic dysfunction-associated fatty liver disease

## Abstract

**Background and Aims:**

Despite the remarkable progress of metabolic dysfunction-associated fatty liver disease (MAFLD), formerly named non-alcoholic fatty liver disease (NAFLD), the disease remains poorly improved. Since increased oxidative stress and inflammation contribute to the initiation and progression of fatty liver disorders, vitamin C (VC), an antioxidant agent, might be a suitable treatment option for MAFLD. However, the lack of clinically confirmed benefits makes clinicians challenging to recommend antioxidant supplements for MAFLD individuals.

**Methods:**

Herein, the nationally representative National Health and Nutrition Examination Survey 2017–2018 data were collected to evaluate the potential association between the serum VC levels with the risk of different categories of NALFD and the newly proposed MAFLD terminology. Hepatic steatosis was defined as controlled attenuated parameter scores ≥ 263 dB/m, whereas liver fibrosis (LF) status was defined as F0–F4, with the cutoff values of median liver stiffness being 6.3, 8.3, 10.5, and 12.5 (KPa), respectively. A cross-sectional analysis was performed to calculate the odds rate and determine the potential beneficial effects of VC.

**Results:**

A total of 4,494 participants aged more than 18 years and conducted transient elastography examinations were included. Our findings demonstrated that participants with increased serum VC status were more likely to be female predominant, more educated, and moderate drinkers. Interestingly, female participants tended to have a lower prevalence of NAFLD, MAFLD, LF, and liver cirrhosis (LC) after stratification by gender. Moreover, our results revealed that participants from the quartile three group (quartile 3: 50.5–67.0 μmol/L) experienced a slightly lower risk of MAFLD than the risk of NAFLD. Of note, the serum concentration of VC (quartile 2: 30.9–50.5 μmol/L) inversely associated with LF and LC was lower than the serum VC level (quartile 3) associated with NAFLD and MAFLD. Notably, individuals from the quartile 3 group experienced a statistically significant 32.5, 42.0, 45.7, and 71% decrease in risk of NAFLD, MAFLD, LF, and LC, respectively.

**Conclusion:**

In summary, our findings suggested an inverse association between serum VC levels and NAFLD, MAFLD, LF, or LC. Additionally, adjustment of VC supplementation according to age, gender, and ethnicity may be a promising candidate for these diseases.

## Introduction

Non-alcoholic fatty liver disease (NAFLD) is a public health problem affecting approximately a quarter of the global population and has been the fastest-growing cause of liver cancer in the United States (1, 2). Despite remarkable progress, this condition remains poorly improved, and effective therapeutic strategies remain elusive. According to the recent consensus, international experts redefined NAFLD as metabolic dysfunction-associated fatty liver disease (MAFLD) *to* establish more clear diagnostic criteria (3). Compared with NAFLD, MAFLD is a broader disease entity that requires the presence of metabolic abnormalities, including obesity and diabetes. The proposed new term from NAFLD to MAFLD is not simply a change to a more appropriate name but also a shift in the populations who meet the criteria for one but not the other. This change highlights the unmet clinical need to investigate the association between promising treatments with those only meeting criteria for MAFLD but not the traditional NAFLD. Accordingly, determining the association of potential treatment strategies with both NAFLD and MAFLD may help to deepen our understanding and application of this new concept (4).

Non-alcoholic fatty liver disease encompasses a continuum of liver disorders, ranging from hepatic steatosis to steatohepatitis (NASH), liver fibrosis (LF), and liver cirrhosis (LC) (5, 6). It is estimated that ~37% of NASH will develop fibrosis, and subsequently, 10–20% of them will develop cirrhosis. Within 5–7 years, 40–60% of cirrhosis can develop into liver failure, and 2.4–12% of cirrhosis eventually progress into hepatocellular carcinoma (HCC) within 3–7 years (7). Although the prognosis is poor, recent studies have shown that mild to moderate LF is reversible, developing after years of NASH with hepatic inflammation. Furthermore, it is generally assumed that the transition from steatosis to NASH is crucial for disease progression, leading to cirrhosis and HCC. For this reason, researchers have focused on steatohepatitis to develop new preventing and reversing strategies. Mechanically, progression from steatosis to NASH and hepatic fibrosis is driven by a series of liver damage resulting from lipid deposition, reactive oxidative species (ROS), nitrogen oxides overload, endoplasmic reticulum stress, and inflammation, which ultimately lead to the activation of hepatic stellate cells, fibrogenesis, and extracellular matrix deposition (8).

In view of the antioxidant function of vitamin C (VC), it could be beneficial in NASH. Previous studies have demonstrated a vicious cycle of deficient balance between oxidant generation and antioxidant defense, leading to liver dysfunctions. Recent studies reported that free fatty acids typically overload in steatosis, resulting in continuous adaptation and further remodeling of structure, mitochondrial bioenergetics, and energy metabolism. Furthermore, the fatty liver tends to be vulnerable to injury, especially when challenged by oxidative stress and lipid peroxidation. The dysfunctional mitochondria in NAFLD are concurrent to incomplete lipid oxidation, leading to the accumulation of lipotoxic lipids, which further activates inflammation, promoting the transition from steatosis to NASH (9–11). Therefore, ROS and inflammation are critical factors in the stepwise progression from simple steatosis to LF and LC. Thus, VC potentially contributes to the alleviation of ROS imbalance and its concomitant pro-inflammatory actions postulated to initiate NASH or cirrhosis.

However, it is difficult for clinicians to recommend the use of antioxidative substances due to the paucity of data on clinically confirmed or definitive physical benefits of VC supplements among patients with MAFLD. Moreover, based on the National Health and Nutrition Examination Survey (NHANES) III data, a recent study found that MAFLD had a greater risk for all-cause mortality, while NAFLD showed no association (3). Hence, assessing the serum VC levels associated with different categories of NAFLD and the proposed term MAFLD may illuminate the potential utility of antioxidative substances between the two entities.

To our knowledge, this is the first study determining the association between serum VC levels with different categories of NALFD and MAFLD using a representative national cohort.

## Methods

### Study Population

**Figure 1:**
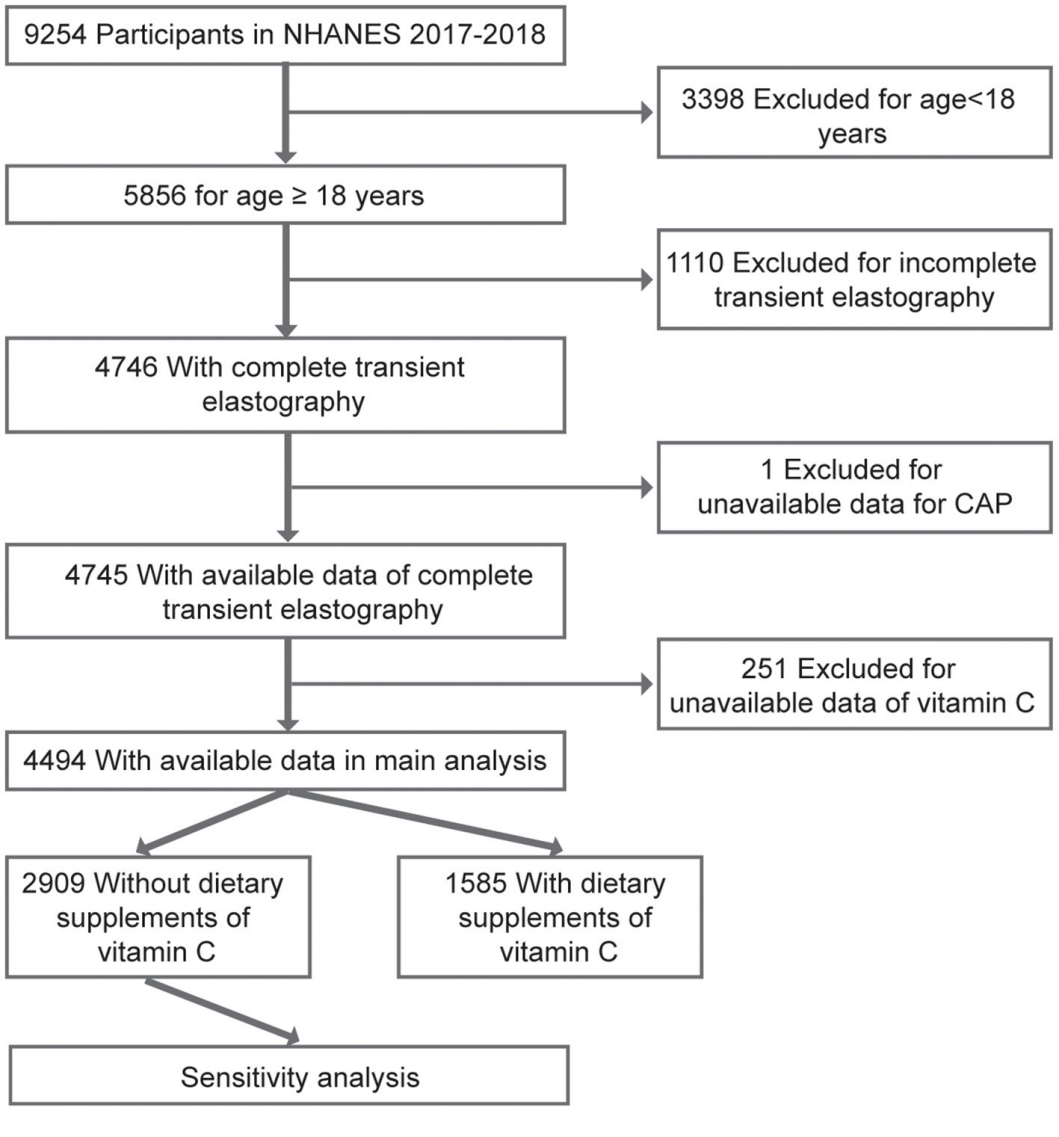
A flowchart showing the selection of study participants. CAP, controlled attenuation parameter.

Data for the current study were collected from NHANES 2017–2018, in which liver ultrasound Transient Elastography (TE) examination was undertaken. NHANES is a nationally representative cross-sectional study designed to examine demographic, socioeconomic, health, and nutrition information. Detailed characterization of NHANES has been reported in previous studies (12). A total of 9,254 participants completed the survey during 2017–2018. However, in the current study, individuals aged <18 years and without complete TE were excluded (*N* = 4,508). In addition, subjects with unavailable data for the controlled attenuated parameter (CAP) or median liver stiffness (LSM) were excluded from the current study (*N* = 1). In addition, participants with missing data on VC were excluded from analysis (*N* = 251). As a result, 4,494 participants were included in the final analysis (Figure 1). Written informed consents were acquired from all study participants and the study protocols were approved by the Research Ethics Review Board of the National Center for Health Statistics. In addition, specific informed consent was not required because of the secondary analysis of public data. The current report was also written based on Strengthening the Reporting of Observational Studies in Epidemiology (STROBE) (13).

### Primary Exposure

During NHANES 2017–2018, participants aged 6 years and older were eligible for serum VC examination. A detailed description of laboratory methodology for serum VC detection has been reported in previous studies (14, 15). A total of 6,740 participants, aged older than 6 years, completed this examination, whereas 695 participants failed to complete the examination. Serum VC levels were categorized into evenly distributed quartiles (<30.9 μmol/L, 30.9–50.5 μmol/L, 50.5–67.0 μmol/L, and ≥67.0 μmol/L).

### Outcomes

Liver ultrasound TE using FibroScan model 502 V2 Touch was first undertaken on NHANES 2017–2018 participants to examine hepatic steatosis and stiffness. TE is a widely used and reliable method to evaluate liver steatosis and fibrosis (16, 17). Participants aged over 12 years old were eligible except for persons (pregnant, could not lie, or had an implanted electronic device/lesion at the examination site). Only subjects with complete tests [fasting time ≥3 h, complete stiffness tests ≥10 measures, and interquartile range (IQR) of liver stiffness/LSM <30%] were included in the current study. Of 4,494 included participants, 3,311 (73.68%) used a medium (M) probe while 1,183 (26.32%) used a large (XL) probe. Herein, hepatic steatosis was defined as CAP scores ≥ 263 dB/m (18), whereas LF status was defined as F0–F4, with the cutoff values of LSM being 6.3, 8.3, 10.5, and 12.5 (KPa), respectively (19).

Non-alcoholic fatty liver disease was diagnosed as the presence of hepatic steatosis without significant alcohol consumption (>3 drinks/day in men and >2 drinks/day in women) and/or viral hepatitis (hepatitis B virus [HBV] or hepatitis C virus [HCV] infections). Individuals with HCV or HBV infections were identified based on positive diagnostic tests (20, 21) or self-reported infection. MAFLD was defined on the basis of steatosis with at least one of the following conditions: (i) body mass index (BMI) ≥ 25 kg/m^2^; (ii) type 2 diabetes which was defined as having a self-reported history of diagnosis with type 2 diabetes or glycohemoglobin ≥ 6.5% (22); (iii) at least 2 of metabolic risk abnormalities below, which included: (i) waist circumference ≥88 cm for women and ≥102 cm for men, (ii) high blood pressure (≥130/85 mmHg) or drug treatment for hypertension, (iii) plasma triglycerides ≥1.70 mmol/L or drug treatment for hyperglyceridemia, (iv) plasma HDL-cholesterol <1.0 mmol/L for men and <1.3 mmol/L for women or drug treatment for hypercholesterolemia, (v) prediabetes (fasting glucose 5.6–6.9 mmol/L or hemoglobin A1c 5.7–6.4%, (vi) homeostasis model assessment of insulin resistance score ≥2.5, and (vii) plasma high sensitivity C-reactive protein (CRP) level >2 mg/L (3, 23). Significant LF and LC were defined as LSM ≥ 6.3 KPa (fibrosis grade ≥ F1) and LSM ≥ 12.5 KPa (fibrosis grade ≥ F4), respectively (19, 24).

### Covariates

In the current study, covariates were ascertained based on known confounders from previously described methods and clinical practice. First, dietary supplements of VC taken from multivitamins or other medications during the past 30 days were considered as “yes/no” variable or in daily (0, 1–60, 61–120, 121–500, or ≥500 mg), or monthly (0, 1–1,800, 1,801–3,600, or ≥3,600 mg) doses (15). The level of dietary VC intake by food was categorized into evenly distributed quartiles (<18.5 g/d, 18.5–47.1 mg/d, 47.1–106.5 mg/d, and ≥106.5 mg/d) and adjusted in the final model. Then, demographic factors including age, gender, and race were selected. The current study classified age into three categories, namely 18–39, 40–59, and 60–80 years. In NHANES 2017–2018, race/ethnicity was classified into Hispanic (such as all Hispanics), non-Hispanic White (such as whites with no Hispanic origin), non-Hispanic Black (such as blacks with no Hispanic origin), non-Hispanic Asian (such as Asians with no Hispanic origin), or other races, including Alaska Natives or American Indians, Native Hawaiians or other Pacific Islanders, and multiracial individuals. Furthermore, BMI (weight/height^2^) was categorized as under/normal weight (<25.0 kg/m^2^), overweight (25.0–30.0 kg/m^2^), and obesity (≥30.0 kg/m^2^).

In addition, alcohol consumption was classified as none, moderate (1 drink/day for women or 1–2 drinks/day for men), heavy (2–3 drinks/day for women or 3–4 drinks/day for men), or binge (≥4 drinks/day for women or ≥5 drinks/day for men) according to definitions from the National Institute on Alcohol Abuse and Alcoholism (NIAAA) in the National Institute of Health. Smoking was classified based on serum cotinine levels into low (<0.015 ng/ml), moderate (0.015–3 ng/ml), and high levels (>3 ng/ml) (25). Moreover, based on Physical Activity Guidelines recommendation of ≥75 min/week of vigorous or ≥150 min/week of moderate physical activity, participants were classified into three groups, namely active (≥the level of recommended activity), less active (< the level of recommended activity), and inactive (no physical activity) (26). The poverty income ratio (ratio of family income to poverty threshold) and was categorized as <1.3, 1.3–1.8, and >1.8. Furthermore, the level of education for participants (more than high school, high school, and less than high school) was also established through interviews.

### Statistical Analysis

Continuous variables were described as weighted mean ± SD and compared using weighted linear regression. Categorical variables were expressed as weighted percentages (95% *CI*) and compared using the chi-square test. Multivariate logistic regression models were constructed to assess the association between VC and NAFLD, MAFLD, LF, or LC. The final model was adjusted for most or all these variables, including gender, age, race, the poverty income ratio, level of education, BMI, serum cotinine levels, daily alcohol consumption, history of diabetes, HBV infection, HCV infection, physical activity status, dietary supplements of VC taken, and level of dietary VC intake by food for different diseases. In addition, subgroup analysis was performed to evaluate the influence of age, gender, race, or BMI on the outcome.

Given that some participants may have high serum VC concentrations because they took high doses of supplements of VC by other multivitamins or medications, we thus adjusted for VC supplementation in the main analysis using the yes/no variable, and daily or monthly doses in a sensitivity analysis. Still for sensitivity analysis, logistic regression was performed again in which we excluded the participants from included subjects who took any additional supplements of VC.

All statistical analyses were undertaken by R software (http://www.R-project.org, The R Foundation, Austria), Empowerstats (http://www.empowerstats.com, X&Y Solutions, Inc, CA, USA), and STATA 16.0 (StataCorp, College Station, TX, USA). Appropriate examination weights were applied to represent the complex survey design. Moreover, two-sided *p* < 0.05 was considered statistically significant.

## Results

### Characteristics of Participants

A total of 4,494 participants were included, of whom 49.15% were male and 50.85% were female, with an average age of 47.13 years old. Overall characteristics of the study subjects by quartiles of serum VC are summarized in Table 1. There were 37.23% NAFLD, 47.98% MAFLD, 21.23% significant LF, and 3.08% LC, among all participants. Statistically significant differences were observed in most outcomes across quartiles of serum VC concentrations, except for HBV infection, aspartate aminotransferase (AST), and total bilirubin (TB) (*p* > 0.05). Besides, those with higher levels of serum VC tended to be female predominant, non-Hispanic White, moderate drinkers, and had the lowest BMI. Subjects with increased serum VC levels were female predominant, more educated, moderate-drinkers, and had the lowest serum cotinine level (<0.015 ng/ml). Conversely, participants with decreased serum VC levels were 40~59 years, male predominant, less educated, and had a higher prevalence of diabetes and obesity. Moreover, the current study observed inverse associations of serum VC levels with the CRP level and prevalence of MAFLD and LF. In contrast, no significant trends were observed for physical activity levels.

**Table 1:** General characteristics of participants (*n* = 4494) stratified by vitamin C (quartiles1-4, umol/L) in the NHANES 2017–2018.

**Characters**	**Total (*n* = 4494)**	**Quartiles 1 (<30.9) (*n* = 1121)**	**Quartiles 2 (30.9–50.5) (*n* = 1124)**	**Quartiles 3 (50.5–67.0) (*n* = 1096)**	**Quartiles 4 (≥67.0) (*n* = 1153)**	***p*-Value**
Age (years)	47.13 ± 17.49	45.94 ± 16.74	45.26 ± 16.81	46.20 ± 17.18	50.73 ± 18.49	<0.001
18~39	38.37 (36.27–40.52)	37.77 (33.67–42.04)	43.13 (38.86–47.50)	40.96 (36.54–45.52)	32.43 (28.63–36.47)	
40~59	34.09 (31.92–36.34)	37.20 (32.88–41.74)	34.44 (30.14–39.00)	33.28 (28.92–37.95)	31.60 (27.48–36.03)	
60~80	27.53 (25.65–29.50)	25.04 (21.62–28.80)	22.43 (19.08–26.18)	25.76 (22.02–29.90)	35.97 (32.01–40.12)	
Gender						<0.001
Men	49.15 (46.93–51.38)	56.73 (52.36–61.00)	54.39 (49.96–58.75)	54.16 (49.57–58.68)	32.74 (28.74–37.00)	
Women	50.85 (48.62–53.07)	43.27 (39.00–47.64)	45.61 (41.25–50.04)	45.84 (41.32–50.43)	67.26 (63.00–71.26)	
Race/ethnicity						<0.001
Hispanic	16.44 (15.27–17.68)	11.94 (10.05–14.12)	21.67 (18.95–24.66)	18.16 (15.70–20.90)	14.61 (12.56–16.93)	
Non-Hispanic White	62.61 (60.71–64.47)	68.11 (64.57–71.44)	52.34 (48.01–56.64)	59.94 (55.84–63.91)	68.74 (65.41–71.88)	
Non-Hispanic Black	10.54 (9.75–11.39)	10.52 (9.07–12.16)	13.43 (11.60–15.49)	10.64 (9.05–12.48)	7.97 (6.70–9.46)	
Non-Hispanic Asian	5.67 (5.18–6.21)	3.48 (2.76–4.38)	7.08 (5.93–8.43)	6.27 (5.26–7.46)	5.99 (5.06–7.08)	
Other races^a^	4.74 (3.91–5.73)	5.96 (4.23–8.35)	5.49 (3.70–8.06)	4.99 (3.45–7.15)	2.69 (1.72–4.19)	
Education						<0.001
More than high school	60.93 (58.80–63.02)	50.49 (46.10–54.88)	59.56 (55.24–63.74)	64.28 (59.93–68.41)	68.93 (65.04–72.57)	
High school or equivalent	27.61 (25.66–29.64)	35.19 (31.08–39.54)	26.48 (22.68–30.66)	26.54 (22.68–30.78)	22.35 (19.02–26.09)	
Less than high school	11.39 (10.40–12.46)	14.31 (12.04–16.94)	13.74 (11.58–16.24)	9.13 (7.55–11.00)	8.68 (7.18–10.46)	
Not recorded	0.07 (0.03–0.16)	-	0.21 (0.07–0.62)	0.05 (0.01–0.21)	0.03 (0.00–0.25)	
Poverty-income ratio						<0.001
<1.3	18.19 (16.85–19.61)	22.48 (19.73–25.49)	19.44 (16.56–22.68)	15.73 (13.25–18.58)	15.32 (13.02–17.94)	
1.3–1.8	8.20 (7.35–9.15)	9.52 (7.70–11.72)	8.24 (6.53–10.33)	8.24 (6.61–10.22)	6.89 (5.51–8.58)	
>1.8	63.57 (61.60–65.51)	57.76 (53.63–61.79)	60.92 (56.72–64.97)	67.96 (64.08–71.61)	67.29 (63.57–70.81)	
Not recorded	10.04 (8.88–11.32)	10.23 (8.07–12.89)	11.40 (8.87–14.55)	8.07 (6.20–10.44)	10.50 (8.32–13.17)	
BMI group						<0.001
<25	27.60 (25.63–29.65)	23.73 (20.23–27.63)	23.31 (19.68–27.37)	25.69 (21.80–30.00)	36.76 (32.65–41.08)	
25–30	31.50 (29.47–33.60)	21.96 (18.65–25.67)	30.99 (27.07–35.19)	39.11 (34.66–43.76)	33.91 (29.93–38.13)	
≥30	40.40 (38.24–42.60)	53.36 (48.97–57.70)	45.34 (40.98–49.78)	34.89 (30.69–39.33)	28.95 (25.25–32.95)	
Not recorded	0.50 (0.33–0.77)	0.96 (0.48–1.88)	0.36 (0.14–0.92)	0.31 (0.14–0.66)	0.38 (0.15–1.01)	
Physical activity level						<0.001
Inactive	50.77 (48.54–53.00)	49.74 (45.34–54.14)	46.94 (42.56–51.36)	49.34 (44.73–53.96)	56.39 (52.03–60.65)	
Less active	7.43 (6.31–8.73)	5.93 (4.27–8.18)	7.94 (5.71–10.94)	8.61 (6.25–11.75)	7.33 (5.30–10.05)	
Active	41.80 (39.61–44.02)	44.33 (40.00–48.75)	45.12 (40.74–49.58)	42.05 (37.56–46.68)	36.28 (32.19–40.58)	
Daily alcohol drinking status						<0.001
Non-drinkers	7.53 (6.50–8.71)	7.49 (5.52–10.08)	6.82 (5.25–8.82)	7.22 (5.18–9.98)	8.48 (6.48–11.03)	
Moderate-drinkers	29.66 (27.58–31.82)	26.48 (22.50–30.89)	27.55 (23.53–31.97)	31.72 (27.43–36.34)	32.58 (28.64–36.79)	
Heavy-drinkers	14.48 (13.00–16.09)	14.77 (11.84–18.27)	15.70 (12.65–19.32)	12.95 (10.37–16.07)	14.57 (11.85–17.79)	
Binge-drinkers	32.79 (30.72–34.94)	34.86 (30.89–39.05)	36.68 (32.46–41.12)	33.61 (29.29–38.22)	26.7 (22.92–30.86)	
Not recorded	15.54 (14.15–17.03)	16.40 (13.62–19.61)	13.25 (11.17–15.64)	14.50 (11.94–17.50)	17.66 (14.69–21.09)	
History of diabetes						<0.001
Yes	12.82 (11.54–14.22)	16.57 (13.68–19.93)	12.87 (10.62–15.51)	11.31 (8.96–14.19)	10.62 (8.43–13.29)	
Having HBV infection						0.105
Yes	0.94 (0.62–1.41)	0.70 (0.39–1.28)	1.31 (0.52–3.27)	1.33 (0.66–2.66)	0.47 (0.25–0.87)	
Having HCV infection						0.011
Yes	2.49 (1.83–3.38)	2.36 (1.57–3.53)	3.09 (1.67–5.64)	1.58 (0.79–3.13)	2.95 (1.52–5.64)	
Dietary VC supplement						<0.001
Yes	38.03 (35.85–40.27)	13.24 (10.29–16.87)	33.25 (29.05–37.74)	40.6 (36.08–45.29)	63.37 (59.19–67.36)	
Daily dose of supplement of VC, mg						<0.001
None	74.68 (72.60–76.65)	94.96 (92.92–96.44)	82.60 (78.57–86.01)	74.21 (69.83–78.16)	48.96 (44.65–53.28)	
1–60	10.08 (8.81–11.52)	2.73 (1.70–4.36)	9.67 (7.21–12.86)	11.48 (8.88–14.72)	16.13 (13.17–19.61)	
60–120	5.03 (4.04–6.26)	0.86 (0.37–2.00)	3.85 (2.28–6.44)	6.71 (4.46–9.96)	8.45 (6.17–11.48)	
121–500	5.62 (4.60–6.84)	0.58 (0.25–1.32)	2.21 (1.01–4.77)	5.29 (3.45–8.05)	13.65 (10.81–17.09)	
>500	4.59 (3.70–5.68)	0.87 (0.34–2.20)	1.66 (0.82–3.34)	2.30 (1.42–3.71)	12.81 (9.97–16.30)	
Monthly dose of supplement of VC, mg						<0.001
None	67.90 (65.74–69.98)	89.95 (86.74–92.45)	71.67 (67.40–75.58)	65.05 (60.46–69.37)	46.31 (42.04–50.63)	
1–1800	15.28 (13.75–16.94)	7.32 (5.18–10.26)	16.58 (13.59–20.09)	17.08 (13.93–20.76)	20.05 (16.85–23.69)	
1800–3600	5.79 (4.75–7.06)	1.55 (0.71–3.36)	6.19 (4.13–9.18)	8.07 (5.59–11.50)	7.38 (5.42–9.96)	
>3600	11.03 (9.66–12.57)	1.18 (0.57–2.42)	5.56 (3.76–8.16)	9.81 (7.42–12.86)	26.27 (22.49–30.43)	
Dietary VC intake by food, mg/d						<0.001
0–18.5	22.73 (20.96–24.61)	36.05 (31.97–40.33)	23.24 (19.63–27.27)	18.21 (15.08–21.82)	13.86 (11.23–16.97)	
18.5–47.1	25.62 (23.66–27.68)	33.68 (29.63–37.99)	29.16 (25.25–33.40)	23.15 (19.16–27.67)	17.19 (14.00–20.94)	
47.1–106.5	24.44 (22.53–26.45)	17.65 (14.52–21.29)	26.48 (22.51–30.86)	24.37 (20.68–28.49)	29.20 (25.31–33.41)	
>106.5	21.68 (19.94–23.52)	8.27 (6.13–11.08)	15.62 (13.08–18.55)	28.66 (24.65–33.03)	33.16 (29.29–37.26)	
Not recorded	5.53 (4.72–6.47)	4.35 (2.81–6.67)	5.51 (4.26–7.11)	5.62 (4.22–7.43)	6.60 (4.88–8.86)	
Laboratory parameters						
Smoking (serum cotinine levels, ng/ml)						<0.001
<0.015	38.17 (35.97–40.42)	27.60 (23.52–32.09)	34.35 (30.23–38.71)	42.38 (37.80–47.09)	47.61 (43.29–51.97)	
0.015–3	37.41 (35.31–39.56)	35.32 (31.24–39.62)	40.29 (36.04–44.69)	36.37 (32.13–40.84)	37.90 (33.88–42.09)	
≥3	24.04 (22.27–25.90)	36.86 (32.89–41.01)	24.47 (20.95–28.37)	20.83 (17.43–24.70)	14.47 (11.77–17.68)	
Not recorded	0.37 (0.15–0.90)	0.23 (0.09–0.58)	0.89 (0.20–4.00)	0.42 (0.18–0.95)	0.01 (0.00–0.10)	
ALT (U/L)	23.26 ± 17.77	25.13 ± 22.03	24.21 ± 17.75	22.14 ± 12.84	21.71 ± 16.99	<0.001
AST (U/L)	22.31 ± 13.41	22.96 ± 18.55	22.15 ± 11.94	22.06 ± 10.16	22.05 ± 11.24	0.304
ALB (g/L)	41.02 ± 3.21	40.36 ± 3.36	40.99 ± 3.26	41.30 ± 3.00	41.40 ± 3.12	<0.001
ALP (U/L)	76.34 ± 25.33	82.37 ± 30.58	77.45 ± 24.94	73.62 ± 21.67	72.19 ± 21.92	<0.001
GGT (U/L)	29.71 ± 40.06	36.16 ± 55.87	34.02 ± 43.07	24.79 ± 25.32	24.46 ± 27.20	<0.001
TC (mmol/L)	4.88 ± 1.04	4.89 ± 1.07	4.92 ± 1.11	4.80 ± 0.97	4.91 ± 1.02	0.017
TB (umol/L)	8.14 ± 4.83	7.89 ± 4.81	8.11 ± 4.62	8.40 ± 4.98	8.17 ± 4.88	0.095
CRP (mg/L)	3.72 ± 7.16	5.14 ± 9.05	3.99 ± 8.43	3.10 ± 5.64	2.72 ± 4.46	<0.001
Platelet ( ×10^∧^9/L)	244.79 ± 61.42	247.07 ± 62.84	248.08 ± 62.13	244.39 ± 63.13	240.15 ± 57.40	0.010
Transient Elastography						
Median stiffness (kPa)	5.66 ± 4.73	6.64 ± 6.79	5.48 ± 3.97	5.34 ± 4.36	5.18 ± 2.71	<0.001
Controlled attenuation parameter (dB/m)	262.47 ± 62.70	277.89 ± 66.53	266.90 ± 60.68	257.71 ± 58.25	248.41 ± 60.94	<0.001
NAFLD						<0.001
Yes	37.23 (35.13–39.39)	43.76 (39.44–48.18)	37.25 (33.21–41.48)	33.91 (29.79–38.28)	34.11 (30.10–38.37)	
MAFLD						<0.001
Yes	47.98 (45.76–50.21)	57.39 (52.98–61.68)	51.24 (46.80–55.66)	44.13 (39.65–48.71)	39.81 (35.63–44.15)	
Liver fibrosis						<0.001
Yes	21.23 (19.48–23.08)	30.58 (26.72–34.74)	20.47 (17.41–23.90)	17.15 (14.01–20.83)	16.79 (13.55–20.62)	
Liver cirrhosis						<0.001
Yes	3.08 (2.35–4.02)	6.41 (4.44–9.16)	2.26 (1.28–3.96)	1.66 (0.98–2.80)	1.93 (0.82–4.51)	

*Values are weighted mean ± SD or weighted % (95% confidence interval). P-values are weighted*.

a *Other races include American Indian or Alaska Native, Native Hawaiian or other Pacific Islander, and multiracial persons*.

*ALT, alanine aminotransferase; AST, aspartate aminotransferase; ALP, alkaline Phosphatase; ALB, albumin; BMI, body mass index; CRP, C reactive protein; GGT, gamma glutamyl transferase; HBV, hepatitis B virus; HCV, hepatitis C virus; MAFLD, metabolic dysfunction-associated fatty liver disease; NHANES, National Health and Nutrition Examination Survey; NAFLD, non-alcoholic fatty liver disease; TC, total cholesterol; TB, total bilirubin; VC, vitamin C*.

Given that our findings on gender predominant are of particular interest, data were further stratified by gender, suggesting a lower prevalence of NAFLD, MAFLD, LF, and LC among women (Supplementary Table 1).

### Associations Between VC and NAFLD or MAFLD

Participants with higher blood VC levels had a decreased risk of NAFLD or MAFLD. Associations of serum VC levels with the risk of NAFLD and MAFLD are presented in Tables 2, 3, respectively. For each model, there were statistically significant associations between VC concentrations and a reduced risk of NAFLD in Q3 [full adjustment, odds ratio (*OR*) = 0.675, 95% *CI*: 0.495–0.920], and MAFLD in Q3–Q4 [full adjustment, Q3: *OR* = 0.580(95% *CI*: 0.434–0.774); Q4: *OR* = 0.490(95% *CI*: 0.362–0.665)]. In fully adjusted models, participants from the Q3 group experienced a 32.5% lower risk for NAFLD and 42% lower risk for MAFLD.

**Table 2:** Associations between serum vitamin C level and NAFLD (*n* = 4494), NHANES 2017–2018.

	**NAFLD (Yes, n = 1802)**	**Model 1 OR (95% CI), *P***	**Model 2 OR (95% CI), *P***	**Model 3 OR (95% CI), *P***
**Quartiles of vitamin C, umol/L**				
Q1 (<30.9)	491	Reference	Reference	Reference
Q2 (30.9–50.5)	485	0.763 (0.593,0.981) 0.035	0.777(0.593,1.019) 0.068	0.756 (0.557,1.027) 0.073
Q3 (50.5–67.0)	444	0.659 (0.508,0.855) 0.002	0.647(0.494,0.846) 0.002	0.675 (0.495,0.920) 0.013
Q4 (≥67.0)	382	0.665 (0.515,0.860) 0.002	0.617(0.470,0.810) 0.001	0.774 (0.556,1.076) 0.128
*P* trend	-	<0.001	<0.001	<0.001
**Sensitivity analysis after exclusion of participants with dietary vitamin C supplement (None**, ***n*** **=** **2909)**				
**Quartiles of vitamin C, umol/L**	*N* = 1136			
Q1 (<30.9)	418	Reference	Reference	Reference
Q2 (30.9–50.5)	332	0.796 (0.600,1.055) 0.112	0.829(0.609,1.130) 0.236	0.789 (0.561,1.111) 0.175
Q3 (50.5–67.0)	247	0.575 (0.423,0.782) <0.001	0.592 (0.433,0.809) 0.001	0.578 (0.403,0.829) 0.003
Q4 (≥67.0)	139	0.510 (0.358,0.727) <0.001	0.541(0.379, 0.771) 0.001	0.683 (0.455,1.024) 0.065
*P* trend	-	<0.001	<0.001	<0.001

*Model 1: Non-adjusted model; Model 2 adjusted for: gender; age; race; Model 3 adjusted for: gender; age; race; education; BMI; diabetes; physical activity status; serum cotinine levels; dietary vitamin C supplement, dietary vitamin C intake by food, and poverty income ratio*.

*NHANES, National Health and Nutrition Examination Survey; BMI, body mass index; NAFLD, non-alcoholic fatty liver disease; OR, odds ratio; 95% CI, 95% confidence interval*.

**Table 3:** Associations between serum vitamin C level and MAFLD (*n* = 4494), NHANES 2017–2018.

	**MAFLD (Yes, *n* = 2230)**	**Model 1 OR (95% CI), *P***	**Model 2 OR (95% CI), *P***	**Model 3 OR (95% CI), *P***
**Quartiles of vitamin C, umol/L**				
Q1 (<30.9)	638	Reference	Reference	Reference
Q2 (30.9–50.5)	611	0.780 (0.607,1.004) 0.053	0.790 (0.603,1.036) 0.088	0.811 (0.613,1.074) 0.144
Q3 (50.5–67.0)	535	0.587 (0.454,0.758) <0.001	0.572 (0.438,0.747) <0.001	0.580 (0.434,0.774) <0.001
Q4 (≥67.0)	446	0.491(0.382,0.632) <0.001	0.478 (0.366,0.624) <0.001	0.490 (0.362,0.665) <0.001
*P* trend	-	<0.001	<0.001	<0.001
**Sensitivity analysis after exclusion of participants with dietary vitamin C supplement (None**, ***n*** **=** **2909)**				
**Quartiles of vitamin C**	*N* = 1455			
Q1 (<30.9)	547	Reference	Reference	Reference
Q2 (30.9–50.5)	424	0.779 (0.589,1.031) 0.081	0.788 (0.582,1.068) 0.125	0.808 (0.593,1.101) 0.177
Q3 (50.5–67.0)	314	0.589 (0.437,0.793) <0.001	0.597 (0.438,0.812) 0.001	0.602(0.434,0.833) 0.002
Q4 (≥67.0)	170	0.391 (0.279,0.549) <0.001	0.421 (0.298,0.595) <0.001	0.420 (0.291,0.607) <0.001
*P* trend	-	<0.001	<0.001	<0.001

*Model 1: Non-adjusted model; Model 2 adjusted for: gender; age; race; Model 3 adjusted for: gender; age; race; education; alcohol; HBV infection; HCV infection; physical activity status; serum cotinine levels; dietary vitamin C supplement, dietary vitamin C intake by food, and poverty income ratio*.

*NHANES, National Health and Nutrition Examination Survey; MAFLD, metabolic dysfunction-associated fatty liver disease; OR, odds ratio; 95% CI, 95% confidence interval*.

Given that 38.03% of participants were VC supplement users, the significant association between blood VC levels and the risk of NAFLD or MAFLD may be explained by dietary VC supplements. Furthermore, according to Table 1, serum VC concentrations were positively associated with dietary VC supplements. To verify this possibility, a sensitivity analysis was performed. Notably, the results remained largely unchanged among participants who did not take VC supplements in all models. In fully adjusted models, similar associations between VC concentrations and a reduced risk of NAFLD [full adjustment, Q3, *OR* = 0.578, 95% *CI*: 0.403–0.829], or MAFLD [full adjustment, Q3, *OR* = 0.602(95% *CI*: 0.434–0.833); Q4, *OR* = 0.420(95% *CI*: 0.291–0.607)] were still present after sensitivity analysis. The conclusions remained unchanged when we further adjusted for daily or monthly doses of VC supplements instead of a yes/no variable. These results of the sensitivity analysis were compatible with the data shown above, further confirming our findings.

As presented in Supplementary Tables 2, 3, subgroup analysis revealed that participants who were among 18~39 years old [full adjustment: NAFLD (Q3, *OR* = 0.541, 95% *CI*: 0.330–0.888; Q4, *OR* = 0.529, 95% *CI*: 0.302–0.927), MAFLD (Q3, *OR* = 0.535, 95% *CI*: 0.352–0.814; Q4, *OR* = 0.342, 95% *CI*: 0.211–0.554)], and non-Hispanic Asian [full adjustment: NAFLD (Q3, *OR* = 0.296, 95% *CI*: 0.149–0.586; Q4, *OR* = 0.197, 95% *CI*: 0.092–0.422); MAFLD (Q3, *OR* = 0.305, 95% *CI*: 0.157–0.590; Q4, *OR* = 0.194, 95% *CI*:0.095–0.397)] had significantly reduced risks of developing both NAFLD and MAFLD in Q3–Q4 groups. After stratifying data by gender, women from Q3 group had a 40.5% reduced risk of NAFLD, while there was no significant association between serum VC levels and NAFLD among men. For Q3 group, an ~50.4% lower and 33.9% lower risk of MAFLD had been found in women and men, respectively. When analyses were stratified by BMI, findings indicated a statistical association of VC with decreased risk of both NAFLD (Q3, *OR* = 0.613, 95% *CI*: 0.399–0.941) and MAFLD (Q3, *OR* = 0.610, 95% *CI*: 0.376–0.989) among participants with BMI ≥ 30 kg/m^2^. Moreover, participants who were among 40–59 years old [full adjustment: Q3 (*OR* = 0.464, 95% *CI*: 0.265–0.814), Q4 (*OR* = 0.516, 95% *CI*: 0.291–0.915)], and Non-Hispanic White [full adjustment: Q3 (*OR* = 0.492, 95% *CI*: 0.325–0.745), Q4 (*OR* = 0.495, 95% *CI*: 0.324–0.756)] also had a significantly reduced risk of developing MAFLD in Q3–Q4 groups.

### Associations Between VC and Significant Fibrosis or LC

The associations of serum VC levels with risks of LF and LC are presented in Tables 4, 5, respectively. In all models and quartiles, inverse associations of VC concentrations and the risk of LF or LC were observed. The fully adjusted *OR*s across quartiles of serum VC concentrations were 1.00 (reference), 0.606 (95% *CI*: 0.451–0.814), 0.543 (95% *CI*: 0.391–0.752), and 0.597 (95% *CI*: 0.400–0.889) for significant LF, and 1.00 (reference), 0.276 (95% *CI*: 0.142–0.534), 0.290 (95% *CI*: 0.139–0.605), and 0.312 (95% *CI*: 0.136–0.717) for LC. Notably, individuals from the Q3 group showed a 45.7% reduced risk of LF and a 71.0% reduced risk of LC (*p* ≤ 0.001). Of note, the serum concentration of VC (Q2: 30.9–50.5 μmol/L) inversely associated with LF and LC was lower than the serum VC level (Q3: 50.5–67.0 μmol/L) associated with NAFLD and MAFLD. Similar and significant results were observed in the sensitivity analysis, except for LF in Q4.

**Table 4:** Associations between serum vitamin C level and significant liver fibrosis (*n* = 4494), NHANES 2017–2018.

	**Significant liver fibrosis (Yes, *n* = 1071)**	**Model 1 OR (95% CI), *P***	**Model 2 OR (95% CI), *P***	**Model 3 OR (95% CI), *P***
**Quartiles of vitamin C, umol/L**				
Q1 (<30.9)	357	Reference	Reference	Reference
Q2 (30.9–50.5)	280	0.584 (0.444,0.769) <0.001	0.590 (0.446,0.780) <0.001	0.606 (0.451,0.814) 0.001
Q3 (50.5–67.0)	229	0.470 (0.346,0.638) <0.001	0.468 (0.344,0.638) <0.001	0.543(0.391,0.752) <0.001
Q4 (≥67.0)	205	0.458 (0.334,0.628) <0.001	0.468 (0.333,0.657) <0.001	0.597 (0.400,0.889) 0.011
*P* trend	-	<0.001	<0.001	<0.001
**Sensitivity analysis after exclusion of participants with dietary vitamin C supplement (None**, ***n*** **=** **2909)**				
**Quartiles of vitamin C, umol/L**	*N* = 705			
Q1 (<30.9)	304	Reference	Reference	Reference
Q2 (30.9–50.5)	184	0.494 (0.362,0.675) <0.001	0.512 (0.373,0.703) <0.001	0.531 (0.379,0.743) <0.001
Q3 (50.5–67.0)	139	0.471 (0.326,0.681) <0.001	0.485 (0.334,0.704) <0.001	0.584 (0.394,0.866) 0.007
Q4 (≥67.0)	78	0.443 (0.274,0.717) 0.001	0.499 (0.304,0.820) 0.006	0.719 (0.414,1.247) 0.240
*P* trend	-	<0.001	<0.001	<0.001

*Model 1: Non-adjusted model; Model 2 adjusted for: gender; age; race; Model 3 adjusted for: gender; age; race; education; alcohol; diabetes; HBV infection; HCV infection; physical activity status; serum cotinine levels; dietary vitamin C supplement; dietary vitamin C intake by food; BMI, and poverty income ratio*.

*NHANES, National Health and Nutrition Examination Survey; BMI, body mass index; OR, odds ratio; 95% CI, 95% confidence interval*.

**Table 5:** Associations between serum vitamin C level and liver cirrhosis, NHANES 2017–2018.

	**Liver cirrhosis (Yes, *n* = 138)**	**Model 1 OR (95% CI), *P***	**Model 2 OR (95% CI), *P***	**Model 3 OR (95% CI), *P***
**Quartiles of vitamin C, umol/L**				
Q1 (<30.9)	61	Reference	Reference	Reference
Q2 (30.9–50.5)	28	0.338 (0.169,0.678) 0.002	0.356 (0.178,0.715) 0.004	0.276 (0.142,0.534) <0.001
Q3 (50.5–67.0)	28	0.247 (0.128,0.477) <0.001	0.251 (0.130,0.483) <0.001	0.290 (0.139,0.605) 0.001
Q4 (≥67.0)	21	0.288 (0.111,0.748) 0.011	0.303 (0.103,0.892) 0.030	0.312 (0.136,0.717) 0.006
*P* trend	-	<0.001	<0.001	<0.001
**Sensitivity analysis after exclusion of participants with dietary vitamin C supplement (None**, ***n*** **=** **2909)**				
**Quartiles of vitamin C, umol/L**	*N* = 85			
Q1 (<30.9)	51	Reference	Reference	Reference
Q2 (30.9–50.5)	14	0.203 (0.094,0.436) <0.001	0.212 (0.101,0.441) <0.001	0.206 (0.095,0.444) <0.001
Q3 (50.5–67.0)	16	0.236 (0.102,0.546) 0.001	0.246(0.108,0.564) 0.001	0.279 (0.095,0.821) 0.020
Q4 (≥67.0)	4	0.108 (0.032,0.358) <0.001	0.112(0.034,0.370) <0.001	0.146 (0.042,0.509) 0.003
*P* trend	-	<0.001	<0.001	<0.001

*Model 1: Non-adjusted model; Model 2 adjusted for: gender; age; race; Model 3 adjusted for: gender; age; race; education; alcohol; diabetes; HBV infection; HCV infection; physical activity status; serum cotinine levels; dietary vitamin C supplement; dietary vitamin C intake by food; BMI, and poverty income ratio*.

*NHANES, National Health and Nutrition Examination Survey; BMI, body mass index; OR, odds ratio; 95% CI, 95% confidence interval*.

In subgroup analysis (Supplementary Table 4), the serum VC level [full adjustment: Q2 (*OR* = 0.561, 95% *CI*: 0.381–0.826), Q3 (*OR* = 0.452, 95% *CI*: 0.291–0.703)] that was inversely associated with LF was relatively lower in men than the VC concentration [full adjustment: Q4 (*OR* = 0.515, 95% *CI*: 0.295–0.902)] in women. Similarly, the concentration of serum VC that was inversely associated with LF among participants with BMI <30 kg/m^2^ [BMI <25 kg/m^2^: (Q3, *OR* = 0.378, 95% *CI*: 0.156–0.919); BMI 25–30 kg/m^2^: (Q3, *OR* = 0.386, 95% *CI*: 0.195–0.766)] was lower than participants with BMI ≥ 30 kg/m^2^ (Q4, *OR* = 0.536, 95% *CI*: 0.323–0.887). The serum concentration of VC statistically associated with LF was lowest in the 18–39 age group [full adjustment: Q2 (*OR* = 0.458, 95% *CI*: 0.286–0.732), Q3 (*OR* = 0.338, 95% *CI*: 0.192–0.594)], intermediate in the 40–59 age group [full adjustment: Q3 (*OR* = 0.516, 95% *CI*: 0.289–0.921)], and highest in the 60–80 age group [full adjustment: Q4 (*OR* = 0.396, 95% *CI*: 0.212–0.740)]. Subgroup analysis of the association between VC levels and cirrhosis were not performed because of the small sample size in that category.

## Discussion

The European Association for the Study of the Liver lifestyle recommended modifications toward a healthy diet and regular exercise for people with NAFLD, while suggested pharmacotherapy should be reserved for people with NASH (27). However, biological complexity and incomplete understanding of NAFLD and MAFLD complicated evidence-based clinical recommendations for VC administration. The present study found that serum VC concentrations were statistically associated with reduced risks of NAFLD, MAFLD, LF, and LC after adjusting for the corresponding risk factors and sensitivity analysis. Of note, the serum concentration of VC inversely associated with LF and LC was lower than the serum VC level associated with NAFLD and MAFLD. Given that the newly proposed MAFLD terminology identified a cohort of individuals with a wider range of metabolic traits, our finding that participants from the Q3 group experienced a slightly lower risk of MAFLD than the risk of NAFLD has major clinical implications.

Our finding that the female sex is associated with a lower prevalence of NAFLD, MAFLD, LF, and LC is of particular interest. Given that the baseline level of VC may impact the benefits of VC administration, sensitivity analysis and gender stratification were conducted. Subsequent analysis revealed that the sex predominant might be partly due to the higher dietary VC supplement among women. Interestingly, a step-like change in the VC concentration associated with LF and LC when stratified by age, suggesting dose adjustment according to age. Another important finding in our study was that participants with obesity and diabetes tended to have lower serum VC levels, which is relevant, as prior studies have shown that NAFLD is particularly common among people with obesity and diabetes (28, 29). The most striking novel finding is the potential hepatoprotective effects of VC, especially for BMI ≥ 30 subjects against NAFLD, MAFLD, and significant fibrosis. These findings are highly important as the epidemic trend of NAFLD has been rising rapidly in recent decades and is increasing in parallel with obesity and diabetes worldwide (28). With a higher burden of metabolic dysregulation, such as obesity and diabetes, it is not surprising that our study found that participants with higher serum VC status had a lower risk of developing MAFLD compared with NAFLD.

It has been reported that the optimum plasma level is about the concentration of saturation (70 μmol/L) (30), which is consistent with our findings that serum VC concentration of 50.5–67.0 μmol/L was associated with decreased risks of NAFLD, MAFLD, LF, and LC. Surprisingly, no correlation was found between the highest quartile of VC and the risk of NAFLD. Additionally, participants in the highest quartile of VC had a slightly higher risk of MAFLD, LF, and LC than those in the 3rd quartile of VC. These data may be partly due to the dual action of VC, which tends to function as a pro-oxidant and contributes to tissue damage at higher concentrations (31, 32). Moreover, several studies have reported that only high doses of VC are associated with liver injury during chronic stress conditions in animal models (33, 34).

Our findings of the inverse association between serum VC levels and a spectrum of liver diseases ranging from MAFLD to LF and LC are in line with prior studies. A recent experimental study has shown that VC treatment decreased high-fat diet-induced NAFLD in mice and had hepatocellular protective effects evidenced by significant weight loss, ballooned hepatocytes, lobular inflammation, and ameliorative liver steatosis (35). To date, research in serum VC levels and NAFLD or MAFLD is scarce, and only two prior studies have found similar associations between VC intake and NAFLD (36, 37). Dana et al. demonstrated that dietary VC intake is inversely associated with lower risks of NAFLD and NASH among 789 subjects (37). However, this analysis might be limited due to the inadequate sample size and inaccurately ultrasonography detection of NAFLD. Furthermore, these findings of dietary VC intake based on recall questionnaires are less reliable due to the absorption obstacles in the gastrointestinal tract, which limited its promising application as a therapeutic agent. Compared with oral VC administration, studies with serum VC levels are often of high quality because circulating VC levels were rarely determined, and therefore, bioavailability and clinical practice could be verified. Notably, we further analyzed the association of serum VC concentrations with the newly proposed MAFLD.

Our novel finding is consistent with previous study findings that VC alleviates inflammation. The subsequent inflammatory environment is a vital contribution to severe NAFLD progression. Several previous studies averred that VC inhibits inflammatory responses mediated by tumor necrosis factor α (TNF-α) and interleukin 6 (IL-6) (38, 39). In addition, studies have indicated that VC potentially reduces inflammatory status through alleviation of CRP and IL-6 (40). Consistent with previous studies, findings of the current study indicated an inverse association between serum VC levels with CRP concentration. Interestingly, Seoung-Woo Lee et al. (41) proposed dual roles of VC in early stages of NAFLD and inflammatory steatohepatitis, and findings indicated that VC deficiency significantly inhibited progression of NAFLD by impairing *de novo* lipogenesis, whereas VC supplementation attenuated inflammatory injuries, including ballooning and lobular inflammation. Therefore, targeted modulation of antifibrotic activity aimed to alleviate the inflammatory environment is a potential therapeutic and preventive strategy against NASH.

Merits of the current study include serum measurement of VC (compared with dietary recall questionnaires) along with representative US civilian data in NHANES. Moreover, this relatively large sample of adults with the TE examination provided opportunities for the study of weak associations (42). The novelty of the present study includes the application of more accurately defined NAFLD using TE compared with an examination of NAFLD using non-invasive algorithms reported in previous studies (43). The current study undertook a detailed stratified analysis, sensitivity analysis, and adjusted for major potential interactions between VC and NAFLD. However, the current study has some limitations. Since the current study adopted a cross-sectional design, temporality cannot be fully clear, which limited the inferences on causes and effects. However, the indicated inverse association between VC with NAFLD is having a reasonable agreement with previous studies on the relationship of VC with fatty liver disease, metabolic syndrome, and inflammation. Another limitation of the current study was the use of TE for diagnosis of NAFLD. Although TE examination is probably the most validated non-invasive method to evaluate liver stiffness (16), the current study lacked histological confirmation. However, TE is considerably an accurate technique, which has been recommended by the World Federation for Ultrasound in Medicine and Biology to distinguish between non-significant and significant fibrosis (44).

## Conclusion

In conclusion, the findings of the current study indicated that increased serum VC concentrations are associated with reduced risks of NAFLD, MAFLD, significant LF and LC. This implies that individuals with MAFLD may benefit from VC supplements. Further studies, including prospective cohort studies, are recommended to identify the clinical significance of VC treatment and prevention of MAFLD.

## Data Availability Statement

Publicly available datasets were analyzed in this study. This data can be found here: https://wwwn.cdc.gov/nchs/nhanes/.

## Author Contributions

Z-QX and H-XL contributed to the conception and design, the acquisition, analysis, interpretation of the data, the drafting of the article, or critical revision for important intellectual content. W-LT, LY, X-WM, W-XL, and Q-BW collected data. C-ZS and Y-JC contributed to the conception and design, the reviewing of the article, or critical revision for important intellectual content. All authors approved the final version and agreed to be accountable for all aspects of the work.

## Funding

W-LT was supported by grant 2020M683094 from the China Postdoctoral Science Foundation and grant 82103221 from the National Natural Science Foundation of China. C-ZS was supported by grant 82072714 from the National Natural Science Foundation of China. Y-JC was supported by grant 81972263 from the National Natural Science Foundation of China and the program of Guangdong Provincial Clinical Research Center for Digestive Diseases (2020B1111170004).

## Conflict of Interest

The authors declare that the research was conducted in the absence of any commercial or financial relationships that could be construed as a potential conflict of interest.

## Publisher's Note

All claims expressed in this article are solely those of the authors and do not necessarily represent those of their affiliated organizations, or those of the publisher, the editors and the reviewers. Any product that may be evaluated in this article, or claim that may be made by its manufacturer, is not guaranteed or endorsed by the publisher.

## Abbreviations

ALT: alanine aminotransferase
AST: aspartate aminotransferase
ALP: alkaline Phosphatase
ALB: albumin
BMI: body mass index
CAP: controlled attenuation parameter
*CI*: confidence interval
CRP: C-reactive protein
GGT: gamma glutamyl transferase
HBV: hepatitis B virus
HCV: hepatitis C virus
IQR: interquartile range
LF: liver fibrosis
LC: liver cirrhosis
LSM: median liver stiffness
MAFLD: metabolic dysfunction-associated fatty liver disease
NAFLD: non-alcoholic fatty liver disease
NHANES: National Health and Nutrition Examination Survey
OR: odds ratio
TC: total cholesterol
TB: total bilirubin
VC: vitamin C.
