# Supplementary material for: Association of Serum Vitamin C With NAFLD and MAFLD Among Adults in the United States
Source: Front Nutr. 2022 Feb 4;8:795391. doi: 10.3389/fnut.2021.795391 (PMC8854786; doi:10.3389/fnut.2021.795391)
Supplement: Supplementary file 1 [file Table_1.pdf]

Supplementary Table 1 General characteristics of participants(n=4494) stratified by gender in the NHANES 2017-2018

| Characters | Total (n=4494) | Male (n=2213) | Female (n=2281) | *p*-Value |
|---|---|---|---|---|
| Age (years) | 47.13±17.49 | 46.24±17.39 | 47.99±17.53 | 0.001 |
| 18~39 | 38.37(36.27-40.52) | 40.26(37.12-43.49) | 36.54(33.79-39.39) | 0.006 |
| 40~59 | 34.09(31.92-36.34) | 34.16(30.98-37.49) | 34.03(31.09-37.10) | |
| 60~80 | 27.53(25.65-29.5) | 25.57(22.99-28.33) | 29.43(26.77-32.24) | |
| Race/ethnicity | | | | 0.782 |
| Hispanic | 16.44(15.27-17.68) | 16.78(15.05-18.66) | 16.11(14.56-17.79) | |
| Non-Hispanic White | 62.61(60.71-64.47) | 62.86(60.07-65.58) | 62.37(59.77-64.90) | |
| Non-Hispanic Black | 10.54(9.75-11.39) | 9.99(8.91-11.19) | 11.07(9.96-12.29) | |
| Non-Hispanic Asian | 5.67(5.18-6.21) | 5.55(4.86-6.35) | 5.79(5.11-6.55) | |
| Other races[1] | 4.74(3.91-5.73) | 4.81(3.64-6.34) | 4.67(3.58-6.06) | |
| Education | | | | 0.010 |
| More than high school | 60.93(58.8-63.02) | 58.47(55.29-61.58) | 63.32(60.45-66.09) | |
| High school or equivalent | 27.61(25.66-29.64) | 29.1(26.23-32.16) | 26.16(23.58-28.91) | |
| Less than high school | 11.39(10.4-12.46) | 12.35(10.86-14.01) | 10.46(9.2-11.88) | |
| Not recorded | 0.07(0.03-0.16) | 0.08(0.02-0.28) | 0.06(0.02-0.17) | |
| Poverty-income ratio | | | | 0.076 |
| <1.3 | 18.19(16.85-19.61) | 16.76(14.92-18.77) | 19.57(17.67-21.62) | |
| 1.3-1.8 | 8.2(7.35-9.15) | 8.14(6.93-9.54) | 8.27(7.1-9.6) | |
| >1.8 | 63.57(61.6-65.51) | 64.51(61.61-67.3) | 62.67(59.95-65.31) | |
| Not recorded | 10.04(8.88-11.32) | 10.6(8.86-12.63) | 9.49(8.04-11.18) | |
| BMI group | | | | <0.001 |
| <25 | 27.6(25.63-29.65) | 23.67(21.04-26.52) | 31.39(28.57-34.36) | |
| 25–30 | 31.5(29.47-33.6) | 34.77(31.71-37.96) | 28.33(25.71-31.11) | |
| ≥30 | 40.4(38.24-42.6) | 40.86(37.67-44.14) | 39.95(37.06-42.91) | |
| Not recorded | 0.5(0.33-0.77) | 0.69(0.4-1.21) | 0.32(0.17-0.59) | |
| Physical activity level | | | | <0.001 |
| Inactive | 50.77(48.54-53) | 44.73(41.51-48.01) | 56.6(53.57-59.58) | |
| Less active | 7.43(6.31-8.73) | 6.56(5.15-8.31) | 8.28(6.64-10.29) | |

| | | | | |
|---|---|---|---|---|
| Active | 41.8(39.61-44.02) | 48.71(45.44-51.99) | 35.12(32.29-38.05) | |
| Daily alcohol drinking | | | | <0.001 |
| Non-drinkers | 7.53(6.5-8.71) | 5.73(4.42-7.41) | 9.27(7.78-11.02) | |
| Moderate-drinkers | 29.66(27.58-31.82) | 32.04(28.98-35.27) | 27.36(24.61-30.29) | |
| Heavy-drinkers | 14.48(13-16.09) | 7.4(5.93-9.21) | 21.32(18.92-23.93) | |
| Binge-drinkers | 32.79(30.72-34.94) | 41.52(38.32-44.79) | 24.36(21.83-27.07) | |
| Not recorded | 15.54(14.15-17.03) | 13.3(11.44-15.42) | 17.7(15.73-19.86) | |
| History of diabetes | | | | 0.069 |
| Yes | 12.82(11.54-14.22) | 13.67(11.77-15.8) | 12(10.34-13.89) | |
| Having HBV infection | | | | 0.237 |
| Yes | 0.94(0.62-1.41) | 1.12(0.61-2.04) | 0.76(0.46-1.26) | |
| Having HCV infection | | | | 0.078 |
| Yes | 2.49(1.83-3.38) | 2.95(1.94-4.47) | 2.04(1.29-3.21) | |
| Dietary VC supplement | | | | <0.001 |
| Yes | 38.03(35.85-40.27) | 33.96(30.81-37.26) | 41.97(38.98-45.01) | |
| Daily dose of supplement of VC, mg | | | | <0.001 |
| none | 74.68(72.6-76.65) | 79.22(76.32-81.86) | 70.28(67.31-73.1) | |
| 1-60 | 10.08(8.81-11.52) | 8.08(6.46-10.06) | 12.02(10.15-14.18) | |
| 61-120 | 5.03(4.04-6.26) | 4.47(3.11-6.39) | 5.58(4.25-7.29) | |
| 121-500 | 5.62(4.6-6.84) | 4.36(3.26-5.82) | 6.82(5.22-8.88) | |
| >500 | 4.59(3.7-5.68) | 3.86(2.69-5.52) | 5.29(4.06-6.87) | |
| Monthly dose of supplement of VC, mg | | | | 0.004 |
| none | 67.9(65.74-69.98) | 69.59(66.37-72.63) | 66.26(63.34-69.06) | |
| 1-1800 | 15.28(13.75-16.94) | 14.61(12.37-17.19) | 15.92(13.93-18.14) | |
| 1800-3600 | 5.79(4.75-7.06) | 6.24(4.59-8.44) | 5.36(4.19-6.83) | |
| >3600 | 11.03(9.66-12.57) | 9.55(7.79-11.65) | 12.46(10.46-14.78) | |
| Dietary VC intake by food, mg/d | | | | <0.001 |
| 0-18.5 | 22.73(20.96-24.61) | 22.44(19.92-25.19) | 23.01(20.58-25.65) | |
| 18.5-47.1 | 25.62(23.66- | 26.20(23.31-29.31) | 25.05(22.46- | |

| | | | | |
|---|---|---|---|---|
| | 27.68) | | 27.83) | |
| 47.1-106.5 | 24.44(22.53-26.45) | 22.23(19.55-25.16) | 26.57(23.92-29.39) | |
| >106.5 | 21.68(19.94-23.52) | 24.01(21.39-26.85) | 19.42(17.21-21.85) | |
| Not recorded | 5.53(4.72-6.47) | 5.11(3.97-6.56) | 5.94(4.85-7.25) | |
| Laboratory parameters | | | | |
| Smoking(serum cotinine levels) | | | | <0.001 |
| <0.015 | 38.17(35.97-40.42) | 33.27(30.14-36.57) | 42.91(39.89-45.98) | |
| 0.015-3 | 37.41(35.31-39.56) | 37.11(34.02-40.31) | 37.71(34.88-40.62) | |
| ≥3 | 24.04(22.27-25.9) | 29.4(26.62-32.33) | 18.87(16.74-21.2) | |
| Not recorded | 0.37(0.15-0.9) | 0.22(0.09-0.53) | 0.52(0.16-1.7) | |
| ALT(U/L) | 47.13±17.49 | 46.24±17.39 | 47.99±17.53 | 0.001 |
| ALB(g/L) | 23.26±17.77 | 27.92±19.64 | 18.73±14.37 | <0.001 |
| ALP(U/L) | 41.02±3.21 | 41.96±3.09 | 40.10±3.06 | <0.001 |
| AST(U/L) | 76.34±25.33 | 76.54±25.67 | 76.15±25.00 | 0.601 |
| GGT(U/L) | 22.31±13.41 | 24.45±14.31 | 20.23±12.12 | <0.001 |
| TB(umol/L) | 29.71±40.06 | 35.61±45.43 | 23.99±33.06 | <0.001 |
| Platelet(×10^9/L) | 8.14±4.83 | 9.5±5.38 | 6.83±3.80 | <0.001 |
| CRP(mg/L) | 244.79±61.42 | 229.79±54.99 | 259.30±63.78 | <0.001 |
| TC(mmol/L) | 3.72±7.16 | 3.1±6.19 | 4.32±7.94 | <0.001 |
| Serum VC(umol/L) | 51.29±29.39 | 46.16±24.9 | 56.25±32.40 | <0.001 |
| <30.9 | 25.39(23.51-27.38) | 29.31(26.43-32.36) | 21.61(19.23-24.19) | |
| 30.9-50.5 | 23.01(21.24-24.87) | 25.46(22.81-28.29) | 20.64(18.35-23.14) | |
| 50.5-67.0 | 24.94(23.01-26.96) | 27.48(24.55-30.61) | 22.48(20.05-25.11) | |
| ≥67.0 | 26.67(24.74-28.69) | 17.76(15.38-20.42) | 35.27(32.41-38.24) | |
| Transient Elastography | | | | |
| Median stiffness(kPa) | 5.66±4.73 | 6.02±4.91 | 5.31±4.52 | <0.001 |
| Controlled attenuation parameter(dB/m) | 262.47±62.7 | 272.47±63.2 | 252.80±60.65 | <0.001 |
| NAFLD | | | | <0.001 |
| Yes | 37.23(35.13-39.39) | 39.9(36.77-43.12) | 34.65(31.86-37.55) | |
| MAFLD | | | | <0.001 |
| Yes | 47.98(45.76-50.21) | 53.56(50.26-56.82) | 42.59(39.64-45.59) | |
| Liver fibrosis | | | | <0.001 |

| | | | | |
|---|---|---|---|---|
| Yes | 21.23(19.48-23.08) | 24.65(22-27.52) | 17.92(15.71-20.35) | |
| Liver cirrhosis | | | | 0.001 |
| Yes | 3.08(2.35-4.02) | 3.92(2.75-5.55) | 2.27(1.5-3.41) | |

Note: Values are weighted mean±SD or weighted % (95% confidence interval). P values are weighted. [1]Other races include American Indian or Alaska Native, Native Hawaiian or other Pacific Islander, and multiracial persons.

Abbreviations: ALT, alanine aminotransferase; AST, aspartate aminotransferase; ALP, alkaline Phosphatase; ALB, albumin; BMI, body mass index; CRP, C reactive protein; GGT, gamma glutamyl transferase; HBV, hepatitis B virus; HCV, hepatitis C virus; MAFLD, metabolic dysfunction-associated fatty liver disease; NHANES, National Health and Nutrition Examination Survey; NAFLD, nonalcoholic fatty liver disease; TC, total cholesterol; TB, total bilirubin; VC, vitamin C.

Supplementary Table 2 Subgroup analysis of associations between serum vitamin C level and NAFLD(n=4494), NHANES 2017–2018.

| **Full adjustment model OR (95%CI), P** | | | | |
|---|---|---|---|---|
| **Quartiles of vitamin C, umol/L** | Q1(<30.9) | Q2(30.9-50.5) | Q3(50.5-67.0) | Q4(≥67.0) |
| **Stratified by age group(years)** | | | | |
| 18-39 | Reference | 0.769(0.477,1.240) 0.281 | 0.541(0.330,0.888) 0.015 | 0.529(0.302,0.927) 0.026 |
| 40-59 | Reference | 0.678(0.407,1.131)0.137 | 0.639(0.367,1.112)0.113 | 0.808(0.451,1.447) 0.473 |
| 60-80 | Reference | 0.838(0.475,1.479)0.542 | 1.085(0.631,1.865)0.769 | 1.051(0.599,1.846)0.861 |
| **Stratified by gender** | | | | |
| Male | Reference | 0.744(0.492,1.125)0.161 | 0.761(0.495,1.168)0.212 | 0.960(0.596,1.548)0.869 |
| Female | Reference | 0.776(0.502,1.200)0.255 | 0.595(0.388,0.912)0.017 | 0.657(0.423,1.021)0.062 |
| **Stratified by race** | | | | |
| Hispanic | Reference | 0.699(0.436,1.121)0.137 | 0.671(0.402,1.118)0.126 | 0.540(0.311,0.938) 0.029 |
| Non-Hispanic White | Reference | 0.691(0.423,1.128)0.139 | 0.595(0.372,0.952)0.031 | 0.837(0.513,1.366)0.477 |
| Non- | Referenc | 1.088(0.665,1.77 | 1.426(0.849,2.39 | 0.640(0.349,1.17 |

| | | | | |
|---|---|---|---|---|
| Hispanic Black | Reference | 9)0.737 | 5)0.179 | 3)0.149 |
| Non-Hispanic Asian | Reference | 0.572(0.301,1.085)0.087 | 0.296(0.149,0.586) <0.001 | 0.197(0.092,0.422) <0.001 |
| Other races[1] | Reference | 0.986(0.328,2.958)0.979 | 0.481(0.163,1.419)0.185 | 1.510(0.427,5.346)0.523 |
| **Stratified by BMI** | | | | |
| <25kg/m$^2$ | Reference | 0.782(0.354,1.724)0.542 | 0.779(0.353,1.717)0.536 | 0.833(0.357,1.944)0.673 |
| 25-30kg/m$^2$ | Reference | 0.849(0.509,1.417)0.532 | 0.799(0.484,1.319)0.380 | 0.712(0.412,1.232)0.225 |
| ≥30kg/m$^2$ | Reference | 0.718(0.477,1.080)0.112 | 0.613(0.399,0.941)0.025 | 0.892(0.549,1.450)0.645 |

Note: Full adjustment model adjusted for: gender; age; race; education; BMI; diabetes; physical activity status; serum cotinine levels; dietary vitamin C supplement, dietary vitamin C intake by food, and poverty income ratio. [1]Other races include American Indian or Alaska Native, Native Hawaiian, or other Pacific Islander, and multiracial persons.

Abbreviations: NHANES, National Health and Nutrition Examination Survey; BMI, body mass index; NAFLD, nonalcoholic fatty liver disease; OR, odds ratio; 95%CI, 95% confidence interval.

Supplementary Table 3 Subgroup analysis of associations between serum vitamin C level and MAFLD(n=4494), NHANES 2017–2018.

| | **Full adjustment model OR (95%CI), P** | | | |
|---|---|---|---|---|
| **Quartiles of vitamin C, umol/L** | Q1(<30.9) | Q2(30.9-50.5) | Q3(50.5-67.0) | Q4(≥67.0) |
| **Stratified by age group(years)** | | | | |
| 18-39 | Reference | 0.956(0.641,1.426)0.826 | 0.535(0.352,0.814)0.003 | 0.342(0.211,0.554) <0.001 |
| 40-59 | Reference | 0.460(0.274,0.774) 0.003 | 0.464(0.265,0.814)0.007 | 0.516(0.291,0.915)0.024 |
| 60-80 | Reference | 1.304(0.761,2.233)0.334 | 0.923(0.544,1.566)0.766 | 0.648(0.370,1.133) 0.128 |
| **Stratified by gender** | | | | |
| Male | Reference | 0.867(0.590,1.274)0.467 | 0.661(0.439,0.994)0.047 | 0.619(0.392,0.978)0.040 |
| Female | Referenc | 0.755(0.505,1.13 | 0.496(0.334,0.73 | 0.411(0.275,0.61 |

| | | | | |
|---|---|---|---|---|
| | e | 1)0.173 | 8)0.001 | 6) <0.001 |
| | | **Stratified by race** | | |
| Hispanic | Reference | 0.813(0.508,1.301)0.388 | 0.664(0.400,1.102)0.113 | 0.389(0.231,0.653)<0.001 |
| Non-Hispanic White | Reference | 0.766(0.499,1.175)0.222 | 0.492(0.325,0.745)0.001 | 0.495(0.324,0.756)0.001 |
| Non-Hispanic Black | Reference | 0.761(0.499,1.160)0.204 | 0.928(0.581,1.482)0.754 | 0.450(0.265,0.763)0.003 |
| Non-Hispanic Asian | Reference | 0.674(0.359,1.267)0.220 | 0.305(0.157,0.590) <0.001 | 0.194(0.095,0.397) <0.001 |
| Other races[1] | Reference | 1.608(0.577,4.476)0.364 | 1.104(0.381,3.199)0.856 | 1.800(0.559,5.801)0.325 |
| | | **Stratified by BMI** | | |
| <25kg/m$^2$ | Reference | 0.685(0.329,1.427)0.312 | 1.231(0.486,3.121)0.661 | 0.979(0.438,2.187)0.959 |
| 25-30kg/m$^2$ | Reference | 1.001(0.606,1.655)0.996 | 0.644(0.393,1.055)0.080 | 0.729(0.432,1.231)0.237 |
| ≥30kg/m$^2$ | Reference | 0.767(0.473,1.243)0.281 | 0.610(0.376,0.989)0.045 | 0.651(0.380,1.112)0.116 |

Note: Full adjustment model adjusted for: gender; age; race; education; alcohol; HBV infection; HCV infection; physical activity status; serum cotinine levels; dietary vitamin C supplement, dietary vitamin C intake by food, and poverty income ratio. [1]Other races include American Indian or Alaska Native, Native Hawaiian or other Pacific Islander, and multiracial persons.

Abbreviations: NHANES, National Health and Nutrition Examination Survey; MAFLD, metabolic dysfunction-associated fatty liver disease; OR, odds ratio; 95%CI, 95% confidence interval.

Supplementary Table 4 Subgroup analysis of associations between serum vitamin C level and significant liver fibrosis(n=4494), NHANES 2017–2018.

| | | **Full adjustment model OR (95%CI), P** | | |
|---|---|---|---|---|
| **Quartiles of vitamin C，umol/L** | Q1(<30.9) | Q2(30.9-50.5) | Q3(50.5-67.0) | Q4(≥67.0) |
| | | **Stratified by age group(years)** | | |
| 18-39 | Reference | 0.458(0.286,0.732)0.001 | 0.338(0.192,0.594) <0.001 | 0.511(0.243,1.075)0.077 |
| 40-59 | Reference | 0.697(0.420,1.155)0.161 | 0.516(0.289,0.921)0.025 | 0.962(0.509,1.818)0.904 |
| 60-80 | Reference | 0.672(0.394,1.147)0.145 | 0.804(0.469,1.377)0.427 | 0.396(0.212,0.740)0.004 |
| | | **Stratified by gender** | | |
| Male | Referenc | 0.561(0.381,0.82 | 0.452(0.291,0.70 | 0.722(0.408,1.27 |

| | | | | |
|---|---|---|---|---|
| | e | 6)0.003 | 3) <0.001 | 9)0.265 |
| Female | Reference | 0.701(0.452,1.087)0.112 | 0.716(0.439,1.167)0.180 | 0.515(0.295,0.902)0.020 |
| **Stratified by race** | | | | |
| Hispanic | Reference | 0.733(0.430,1.248)0.252 | 0.506(0.280,0.916)0.024 | 0.509(0.253,1.025)0.059 |
| Non-Hispanic White | Reference | 0.449(0.276,0.732)0.001 | 0.502(0.304,0.827)0.007 | 0.590(0.329,1.059)0.077 |
| Non-Hispanic Black | Reference | 1.440(0.905,2.292)0.124 | 1.264(0.757,2.112)0.370 | 0.992(0.546,1.805)0.980 |
| Non-Hispanic Asian | Reference | 0.499(0.236,1.055)0.069 | 0.342(0.153,0.766)0.009 | 0.435(0.196,0.964)0.040 |
| Other races[1] | Reference | 0.515(0.148,1.789)0.296 | 0.396(0.114,1.384)0.147 | 0.190(0.040,0.900)0.036 |
| **Stratified by BMI** | | | | |
| <25kg/$m^2$ | Reference | 0.677(0.361,1.270)0.225 | 0.378(0.156,0.919)0.032 | 0.603(0.185,1.971)0.403 |
| 25-30kg/$m^2$ | Reference | 0.715(0.393,1.302)0.273 | 0.386(0.195,0.766)0.006 | 0.604(0.313,1.166)0.133 |
| ≥30kg/$m^2$ | Reference | 0.567(0.386,0.833)0.004 | 0.748(0.484,1.158)0.193 | 0.536(0.323,0.887)0.015 |

Note: Full adjustment model adjusted for: gender; age; race; education; alcohol; diabetes; HBV infection; HCV infection; physical activity status; serum cotinine levels; dietary vitamin C supplement; dietary vitamin C intake by food; BMI, and poverty income ratio. [1]Other races include American Indian or Alaska Native, Native Hawaiian or other Pacific Islander, and multiracial persons.

Abbreviations: NHANES, National Health and Nutrition Examination Survey; BMI, body mass index; OR, odds ratio; 95%CI, 95% confidence interval.
